# Distributed causality in resting-state network connectivity in the acute and remitting phases of RRMS

**DOI:** 10.1186/s12868-020-00590-4

**Published:** 2020-09-15

**Authors:** Lin Wu, Muhua Huang, Fuqing Zhou, Xianjun Zeng, Honghan Gong

**Affiliations:** 1 Department of Radiology, The First Affiliated Hospital, Nanchang University, Nanchang, Jiangxi People’s Republic of China; 2 Jiangxi Province Medical Imaging Research Institute, Nanchang, Jiangxi People’s Republic of China

**Keywords:** Acute phase, Remitting phase, Multiple sclerosis, Resting-state networks, Granger causality

## Abstract

**Background:**

Although previous studies have shown that intra-network abnormalities in brain functional networks are correlated with clinical/cognitive impairment in multiple sclerosis (MS), there is little information regarding the pattern of causal interactions among cognition-related resting-state networks (RSNs) in different disease stages of relapsing–remitting MS (RRMS) patients. We hypothesized that abnormalities of causal interactions among RSNs occurred in RRMS patients in the acute and remitting phases.

**Methods:**

Seventeen patients in the acute phases of RRMS, 24 patients in the remitting phases of RRMS, and 23 appropriately matched healthy controls participated in this study. First, we used group independent component analysis to extract the time courses of the spatially independent components from all the subjects. Then, the Granger causality analysis was used to investigate the causal relationships among RSNs in the spectral domain and to identify correlations with clinical indices.

**Results:**

Compared with the patients in the acute phase of RRMS, patients in the remitting phase of RRMS showed a significantly lower expanded disability status scale, modified fatigue impact scale scores, and significantly higher paced auditory serial addition test (PASAT) scores. Compared with healthy subjects, during the acute phase, RRMS patients had significantly increased driving connectivity from the right executive control network (rECN) to the anterior salience network (aSN), and the causal coefficient was negatively correlated with the PASAT score. During the remitting phase, RRMS patients had significantly increased driving connectivity from the rECN to the aSN and from the rECN to the visuospatial network.

**Conclusions:**

Together with the disease duration (mean disease duration < 5 years) and relatively better clinical scores than those in the acute phase, abnormal connections, such as the information flow from the rECN to the aSN and the rECN to the visuospatial network, might provide adaptive compensation in the remitting phase of RRMS.

## Background

Multiple sclerosis (MS) is a chronic inflammatory demyelination disease of the central nervous system that can cause progressive neurological deficits in young adults, including up to 70% cognitive impairment [1]. Developments in functional magnetic resonance imaging (fMRI) could help to further unravel the underlying mechanisms that cause these cognitive deficits [2]. Notably, fMRI data have been used to examine the functional connectivity (FC) of spatially remote brain regions to delineate a set of functional networks that exhibit high reproducibility and moderate to high test–retest reliability [3–5]. Some studies in MS have found that the default mode network (DMN), which is associated with internally directed cognition, exhibits decreased activation during cognitive task performance [6] and disturbed FC within DMN regions during rest [7–9]. Additionally, patients with MS with cognitive impairment display reduced eigenvector centrality dynamics in the DMN, frontoparietal network, and visual network [10]. Our previous study found that abnormalities of the attention network, which is more associated with externally directed cognition, have been observed in relapsing–remitting MS (RRMS); these abnormalities include decreased FC [11] and dynamic FC [12] within the network. Overall, previous studies have demonstrated that extensive functional networks present dysfunction, which is related to the cognitive performance in MS patients.

To better understand the cognitive functions of the human brain, it is essential not only to study individual or intrinsic functional networks but also to consider how they work together as a whole. A functional network connectivity study found that abnormalities of functional interactions between the principal resting-state networks (RSNs) were also present in RRMS patients and were related to the severity of the disability [13]. Recently, a few studies have shown abnormal functional connection strength among the DMN, attention network and working memory network in paediatric MS [14] and the maintenance of a stable interaction between the DMN and attention network in adult RRMS patients in the remitting phase [11]. Moreover, previous MS studies indicated that structural brain damage might result in functional reorganization (increased FC) before the functional network collapses, prompting a delayed cognitive decline [15]. However, either an increase or decrease in the FC of the internetwork correlates with a decline in cognition [9, 15–17]. In truth, we are currently unable to simplistically think whether the increased functional connection is good or bad. Additionally, previous studies did not account for causal interactions among cognition-related RSNs in different stages of disease in patients with RRMS. Granger causality analysis (GCA) provides a powerful and generic statistical tool for characterizing directed functional interactions from time-series data. Yan et al. [18] found the GCA-based functional directed network of the human brain is the stability and reproducibility and suggested the GCA might be a reliable approach for the performance of a spontaneous causal influence analysis with resting-state fMRI.

Therefore, we hypothesized that abnormalities in the causal interactions among RSNs occurred in RRMS patients in the acute and remitting phases. We first used group independent component analysis (ICA) to extract RSN time courses in patients with RRMS and healthy controls and identified 14 consistent large-scale RSNs through a voxelwise correlation with a template-matching algorithm [19]. Then, to quantify the strength of interactions and to reveal directed interactions between independent components, the GCA was used to investigate interactions among fourteen RSNs. Connectivity was compared between patients experiencing the acute phase of RRMS and healthy controls and between patients experiencing the remitting phase of RRMS and healthy controls. Subsequently, we assessed the correlation between altered causal coefficients and the clinical profile in RRMS. This study may help our understanding of the correlation between brain functional network rewiring and clinical dysfunction in RRMS.

## Materials and methods

### Subjects

From May 2014 to December 2018, a total of 41 patients with clinically diagnosed RRMS (17 in acute phases and 24 in remitting phases) were recruited at the First Affiliated Hospital of Nanchang University, according to the 2010 revised McDonald’s criteria [20]. Twenty-three appropriately matched (age, sex, and education) subjects served as healthy controls (HCs). The exclusion criteria for the subjects were the presence or history of traumatic brain injuries, tumour or stroke based on conventional MRI data. All subject have self-reported being right-handed.

All the patients underwent neuropsychological evaluations, including the Expanded Disability Status Scale (EDSS), Modified Fatigue Impact Scale (MFIS), and Paced Auditory Serial Addition Test (PASAT).

### Image acquisition and preprocessing

All the subjects were imaged with a 3.0 T MRI scanner (Trio Tim; Siemens, Munich, Germany) using an eight-channel phased array head coil. The following sequences of the brain were acquired: (1) T2*-weighted gradient echo sequence (TR/TE = 2,000/30 ms, flip angle = 90°, FOV = 200 × 200 mm, matrix = 64 × 64, 30 interleaved axial slices with a 4 mm thickness and an interslice gap of 1.2 mm, number of time points = 240); (2) T2-weighted turbo spin-echo imaging; and (3) three-dimensional T1-weighted imaging. During the RS-fMRI scanning, the subjects were instructed simply to rest with their eyes closed, not to think systematically, and not to fall asleep.

All the preprocessing steps were carried out using the MATLAB 2012a platform (MathWorks, Inc., Natick, MA, USA). The standard preprocessing procedures have been described in our previous studies [11]. The main steps of preprocessing included the first ten volumes of each session were discarded for the equilibrium state of the echo signal, slice correction, head realignment, spatial normalization to MNI space with high-resolution T1WI registration, resampling to 3 mm isotropic voxels, and 6 mm smoothing. Besides, subjects with head movement in the cardinal directions (x, y, z) > 2 mm and a maximum rotation (x, y, z) > 2° were excluded.

### Group ICA

We performed a group spatial ICA on the preprocessed data of the patients with RRMS and normal controls using the Group ICA of fMRI Toolbox (GIFT, https://icatb.sourceforge.net/groupica.htm). We chose a relatively high model order ICA (number of components, C = 75), as previous studies have demonstrated that such models yield refined components [21] and a highly stable ICA decomposition [22]. In the group ICA, the mean independent components of all the subjects, the corresponding mean time courses and the independent components for each subject were obtained from the group ICA separation and back reconstruction to ensure that all the subjects had the same components [23]. After standard preprocessing of the group ICA results, from 75 components, we identified fourteen RSNs via a template-matching algorithm based on the maximum spatial correlation value. These functional templates were provided by Shirer et al. [19]. Then, one-sample t-test of the group-wise spatial maps was performed by Data Processing Assistant for Resting-State fMRI Advanced Edition (version 2.2).

### GCA

The GCA in a spectral method that is used to elucidate the causal relationships between two or more stationary variables for brain networks [24]. Based on the principle of Granger causality, if incorporating the past values of time series X improves the future prediction of time series Y, then X is said to have a causal influence on Y [25]. Granger causality analysis was accomplished using the functional network connectivity toolbox (https://icatb.sourceforge.net/). According to a given interval and an order selection criterion, the optimal order of the autoregressive model was selected, which was not constant and varied within the interval for every mutual relationship. We preferred the Schwartz Bayesian criterion to determine the optimal order of the autoregressive model and to obtain the smallest mean-squared prediction error of the fitted autoregressive model. In this study, the GCA was compared between the acute phase of RRMS and healthy controls and between the remitting phase of RRMS and healthy controls. The statistical significance level was set at a *P*-value less than 0.05 with false discovery rate (FDR) correction.

### Statistical analysis

We performed statistical analyses of the demographic, clinical, and relationship data by using SPSS software (version 22, 2013; IBM, Chicago, Ill). We investigated Pearson’s correlation between abnormal Granger causality coefficients and neuropsychological characteristics in RRMS while controlling for age and sex. The relationship was significant if the *P*-value was below 0.05.

## Results

### Demographic and clinical data

Table 1 shows the demographic and clinical characteristics of the RRMS patients and HCs. Significant differences in the EDSS, PASAT and MFIS were observed between the acute phase and remitting phase of RRMS.

**Table 1:** Demographic and clinical characteristics of the study population

	RRMS		HCs (n = 23)	*P*-values^a^	*P*-values^b^	*P*-values^c^
	Acute phase (n = 17)	Remitting phase (n = 24)				
Gender (M/F)	7/10	9/15	12/11	0.504	0.322	n/a
Mean age (range) (years)	43.1 (15–61)	40.7 (21–66)	40.7 (26–58)	0.446	0.996	n/a
Mean disease duration (range) (months)	23.3 (2–55)	31.1 (4–150)	–	n/a	n/a	n/a
Mean EDSS (range)	2.8 (1.5–4)	2.0 (0–3.5)	–	n/a	n/a	0.015
Mean PASAT (range)	88.3 (76–103)	90.2 (80–103)	–	n/a	n/a	0.002
Mean MFIS (range)	11.2 (6–16)	9.0 (2–15)	–	n/a	n/a	0.03

^a^Comparison between the acute phase of RRMS and healthy controls

^b^Comparison between the remitting phase of RRMS and healthy controls

^c^Comparison between the acute phase and remitting phase of RRMS

### Spatial distribution of RSNs

**Fig. 1:**
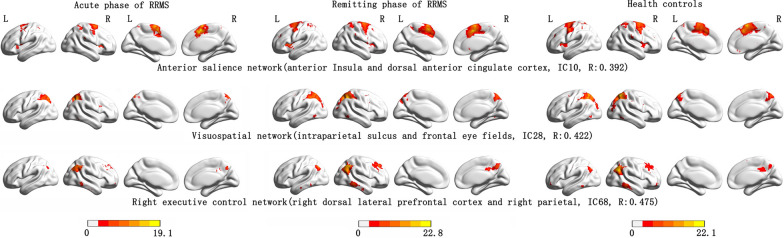
Spatial map of the three resting state networks from relapsing–remitting multiple sclerosis and health controls. (one-sample *t*-test, P = 0.001, FDR correction). IC 10: independent component 10 obtained by group independent components analysis. R is the highest correlation coefficient which results from the spatial match of the independent components with the offering templates

An analysis of the fMRI data revealed 14 spatial maps of potentially relevant RSNs, including the posterior salience network (IC 8), anterior salience network (aSN) (IC 10), basal ganglia network (IC 20), primary visual network (IC 23), visuospatial network (IC 28), higher visual network (IC 58), language network (IC 59), sensorimotor network (IC 62), auditory network (IC 63), left executive control network (IC 66), right executive control network (rECN) (IC 68), dorsal DMN (IC 70), praecuneus network (IC 74), and ventral DMN (IC 75). (Fig. 1 and Additional file 1: Fig. S1).

### Causal relationships of RSNs

**Fig. 2:**
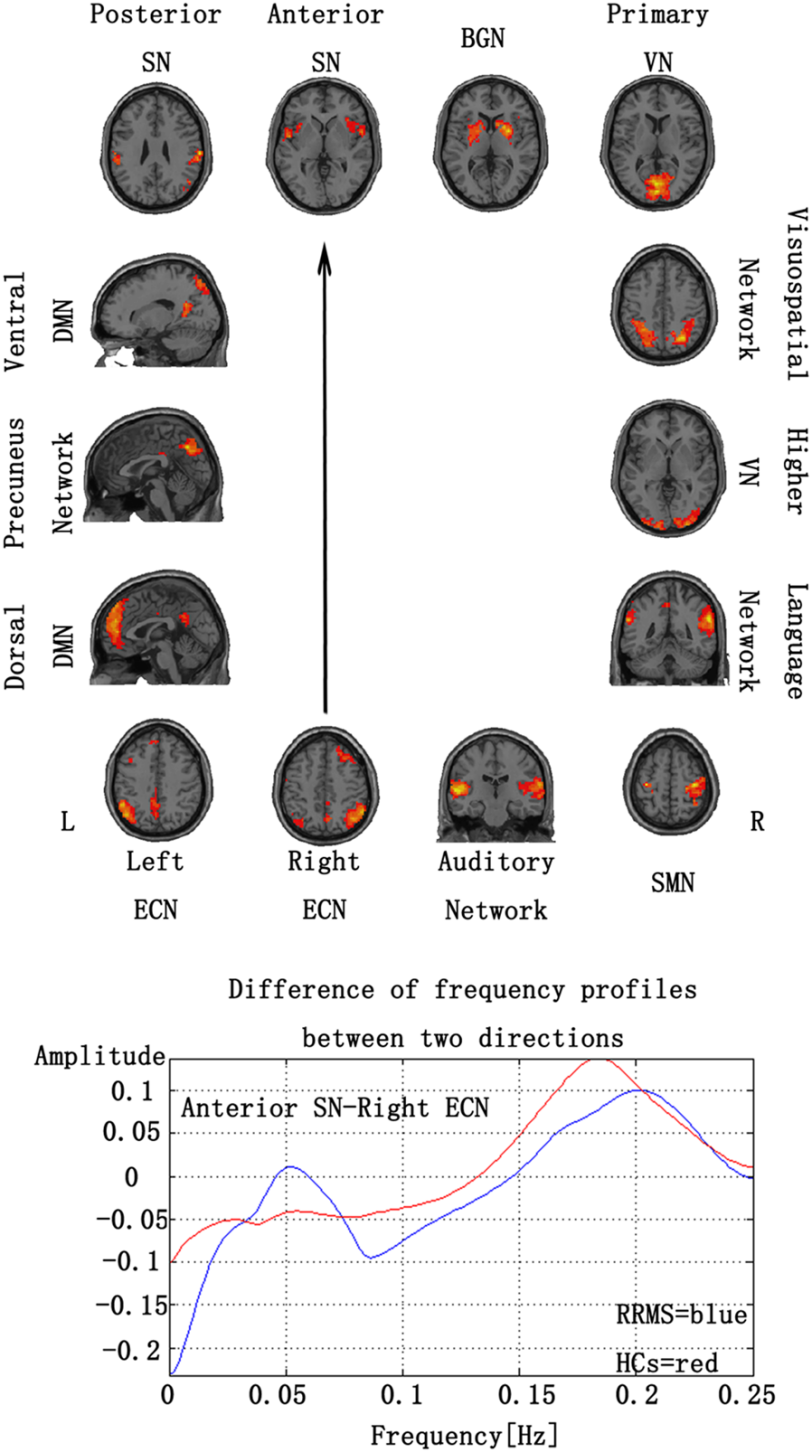
Group differences in the GCA in the spectral domain between the acute phase RRMS patient and HCs. (P < 0.05, FDR correction). The positive value of amplitude means the direction from the anterior SN to the right ECN. On the contrary, the negative value means the direction from the right ECN to the anterior SN

**Fig. 3:**
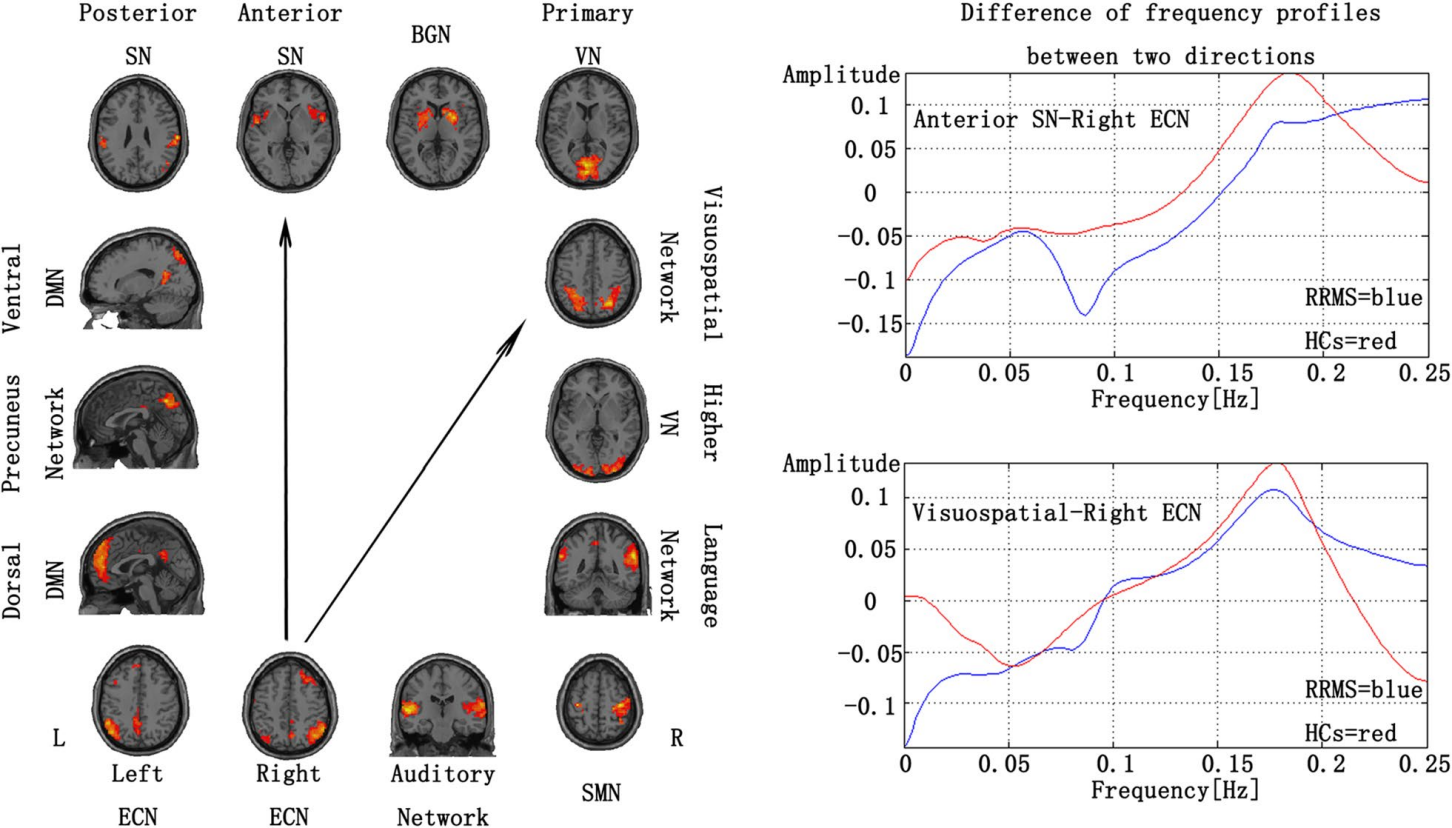
Group differences in the GCA in the spectral domain between the remitting phase RRMS patient and HCs. (P < 0.05, FDR correction). The positive value of amplitude means the direction from the anterior SN to the right ECN. On the contrary, the negative value means the direction from the right ECN to the anterior SN

We also used the GCA to investigate the causal relationships of fourteen RSNs compared between the RRMS and HCs. During the acute phase of RRMS, the patients had a significantly increased driving connectivity from the rECN to the aSN. (Fig. 2 and Additional file 1: Fig. S2). During the remitting phase of RRMS, the patients had significantly increased driving connectivity from the rECN to the aSN and from the rECN to the visuospatial network. (Fig. 3 and Additional file 1: Fig. S3) (P < 0.05, FDR correction).

### Analysis of correlations

**Fig. 4:**
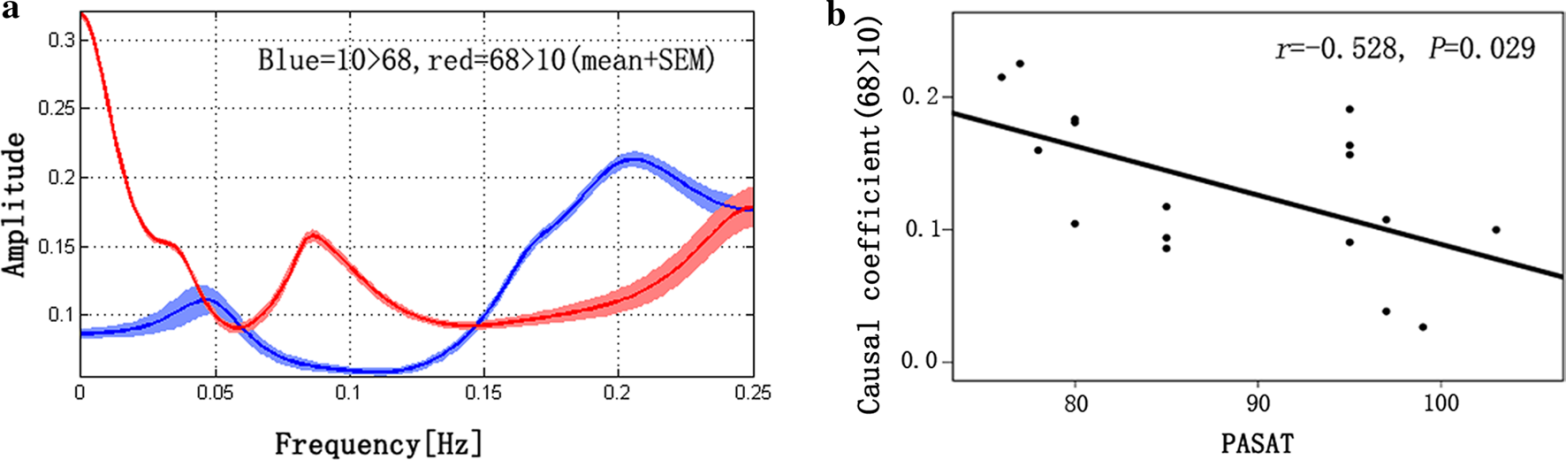
Correlations between the PASAT score and the altered connectivity coefficients in the acute phase of RRMS. **a** Frequency profiles for both directions; **b** Pearson’s correlation scatter plot. 68 > 10 means causal coefficient from component 68 (right executive control network) to component 10 (anterior salience network)

Our study only observed that an increased causal coefficient from the rECN to the aSN was negatively correlated with the PASAT score in the acute phase of RRMS (*r* = − 0.528, *P* = 0.029). The other abnormal causal coefficients did not show significant correlations with the clinical parameters (including disease duration, EDSS, PASAT and MFIS) (Fig. 4).

## Discussion

We applied group ICA and Granger causality to reveal relationships among fMRI brain networks in the spectral domain between RRMS patients and HCs. Depending on the different stages of the disease, patients with RRMS were observed to have different network causal connections. These results represent the general direction of the information flow. This approach investigating the direction of causality among cognition-related RSNs can form a useful complement to aid in understanding the underlying compensatory mechanism in MS.

### Comparison between patients in the acute phase of RRMS and HCs

ECN and aSN dysfunctions have been described in MS [13, 26]. The ECN is functionally related to but dimensionally separate from the aSN. While the ECN is thought to be involved in goal-directed behavior and perceptual processes [27], the primary roles of the aSN are associated with stimulus-driven processes, detecting salient cues in the environment, interrupting ongoing activity in the ECN [28] and coordinating neural resources [29, 30]. The FC between these two networks has been described to guide interactions with the external environment and responses to potentially threatening stimuli [31]. Moreover, a study of the Stroop interference task showed that the rECN had the greatest number of outgoing connections and sent information to the insula (the key nodes of the SN), and the study noted that the abnormal interaction of MS patients might correlate with deficits of cognitive performance [32]. Our results represent the general direction of the information flow from the rECN to the aSN in the acute phase of RRMS in task-free conditions. Interestingly, we found that an increased causal coefficient was negatively correlated with the PASAT score. Historically, most early studies have used the PASAT to study cognition in MS, as it measures sustained attention and information processing speed [33, 34]. Although this neural mechanism facilitates access to resources that engage the PASAT task by activating the rECN [6, 35], one should exercise caution regarding the interpretation of how causal changes may underlie clinical and cognitive deterioration in MS. Future longitudinal assessments will provide the opportunity to define whether an abnormal causal relationship has a role in the changes in cognitive function in the acute phase of RRMS.

### Comparison between patients in the remitting phase of RRMS and HCs

Compared with HCs, we also observed significantly increased driving connectivity from the rECN to the aSN and the visuospatial network in patients in the remitting phase of RRMS. Our partial result is consistent with a previous functional network connectivity study in which the ECN had increased connectivity with the SN in the remitting phase of RRMS in task-free conditions [13]. However, no previous studies have reported driving connectivity from the rECN to the visuospatial network in the remitting phase of RRMS. The visuospatial network, whose core regions include the intraparietal sulcus and the frontal eye field, is related to a specific modulation of corresponding locations of visual perception [36]. The visuospatial network regions send top-down biases to visual areas via the middle frontal gyrus (a core region of the ECN) to the ventral attention system, the core regions of which include the anterior insula, restricting ventral activation to behaviorally important stimuli. Conversely, when attention is reoriented to a new source of information (stimulus-driven reorienting), the ventral attention system sends a reorienting signal to the visuospatial network through the middle frontal gyrus (as a circuit breaker), which shifts attention towards the novel object of interest [37, 38]. In summary, neuroimaging evidence suggests that when novel stimuli involving cognition and emotion are present, the interaction among the visuospatial network, the ECN and the SN would form the control systems of the brain to respond stimuli [31, 39].

Recent studies also show that shorter and longer RRMS disease duration with similar disabilities are characterized by distinct patterns of FC, involving predominantly sensory and cognitive networks, respectively [40]. Besides, increased FC between brain regions implies the use of brain reserves to ameliorate cognitive impairment in early-stage MS patients (disease duration < 5 years), which is generally interpreted as a compensatory mechanism [34, 41]. During the course of disease after reaching a maximal level, brain FC enhancement decreases and decreased FC participates in disability progression, according to a longitudinal resting-state fMRI study [42]. Moreover, an anatomo-functional study revealed that a brain network disconnection may deprive the brain of compensatory mechanisms in cognitively preserved patients with early MS (3–5 years disease duration) [8]. Taken together, these studies suggest that in patients with shorter disease duration (mean disease duration < 5 years) and relatively better clinical scores (EDSS, PASAT, MFIS) in the remitting phase compared to those in the acute phase of RRMS, increased driving connectivity from the rECN to the aSN and from the rECN to the visuospatial network may be an adaptive compensatory mechanism to limit the clinical consequences of disease-related tissue damage.

In the end, our study shows opposite directions of causal association between low and high frequency ranges. For example, in Fig. 2, the high frequency range is indicated as the direction from the aSN to the rECN. On the contrary, low frequency range means the direction from the rECN to the aSN. Directional relationships in low and high frequencies could implicate different causal associations. Causality in a low frequency could suggest that a sustained state was alternated to the other state in the identified order. In contrast, causality in a high frequency could suggest a transfer of frequent transient activation between the networks. Although the order of activations in a short interval is hard to identify due to a short TR (2-s) and variability of the hemodynamic response function, a slow network alteration in low-frequency range can be validated. The part of low-frequency caused the difference between the groups in our study (showing Additional file 1: Figs. S2, S3). Future researches about Granger prediction between different frequency bands will give us more useful insights into the pathology of RRMS.

## Limitations

First, altered FC is related to the level of structural damage and distribution of MS lesions [9, 12], and it has also been characterized by mostly heterogeneous findings. For instance, the consensus is that MS lesions disrupt the corresponding white matter pathways, leading to dysfunction. However, recent research has suggested that physical and cognitive disabilities seem to correlate better with damage to grey matter than with the lesion load of white matter in MS patients [43]. Future research is needed to elucidate the association between structural damage (grey matter and white matter) and causal changes involving cortical and subcortical RSNs in MS. Second, it should be noted that the observed correlation in the acute phase was relatively weak, and no correlations were observed in the remitting phase of RRMS. Undeniably, our acquired images (4 mm slice thickness and 2-s TR) limited the spatial and time resolution research on this “causality” to a certain extent. Thus, higher-resolution fMRI data (especially with shorter TR scanning) studies should be conducted in the future. Third, the result of correlation analysis (P = 0.029) cannot survive the correction for testing multiple scales. Therefore, a greater number of subjects should be used in future studies.

## Conclusion

In summary, we found abnormalities in causal interactions between cognition-related RSNs in patients in the acute phases and remitting phases of RRMS compared with HCs. These findings, together with the disease duration and relatively better clinical scores in patients in the remitting phase than in the acute phase, highlight the complexity of the interactions between RSNs that might act as adaptive compensatory mechanisms in the remitting phase of RRMS. A longitudinal study in the future may help to determine the extent of connectivity changes between the resting state networks with the progression of the disease.

## Supplementary information

Additional file1 (DOCX 1556 kb)

## Data Availability

All raw data are stored in Department of Radiology, The First Affiliated Hospital, Nanchang University. The datasets analyses are available from the corresponding author on reasonable request.

## Abbreviations

MS: multiple sclerosis
fMRI: functional magnetic resonance imaging
FC: functional connectivity
DMN: default mode network
RRMS: relapsing–remitting MS
RSNs: resting-state networks
ICA: independent component analysis
GCA: granger causality analysis
HCs: healthy controls
EDSS: disability status scale
MFIS: modified fatigue impact scale
PASAT: paced auditory serial addition test
TR: repetition time
TE: echo time
FDR: false discovery rate
aSN: anterior salience network
BGN: basal ganglia network
rECN: right executive control network
VN: visual network
SEM: standard error of mean
