# Supplementary material for: Distributed causality in resting-state network connectivity in the acute and remitting phases of RRMS
Source: BMC Neurosci. 2020 Sep 15;21:37. doi: 10.1186/s12868-020-00590-4 (PMC7493168; doi:10.1186/s12868-020-00590-4)
Supplement: Supplementary file 1 — Additional file1 (DOCX 1556 kb) [file 12868_2020_590_MOESM1_ESM.docx]

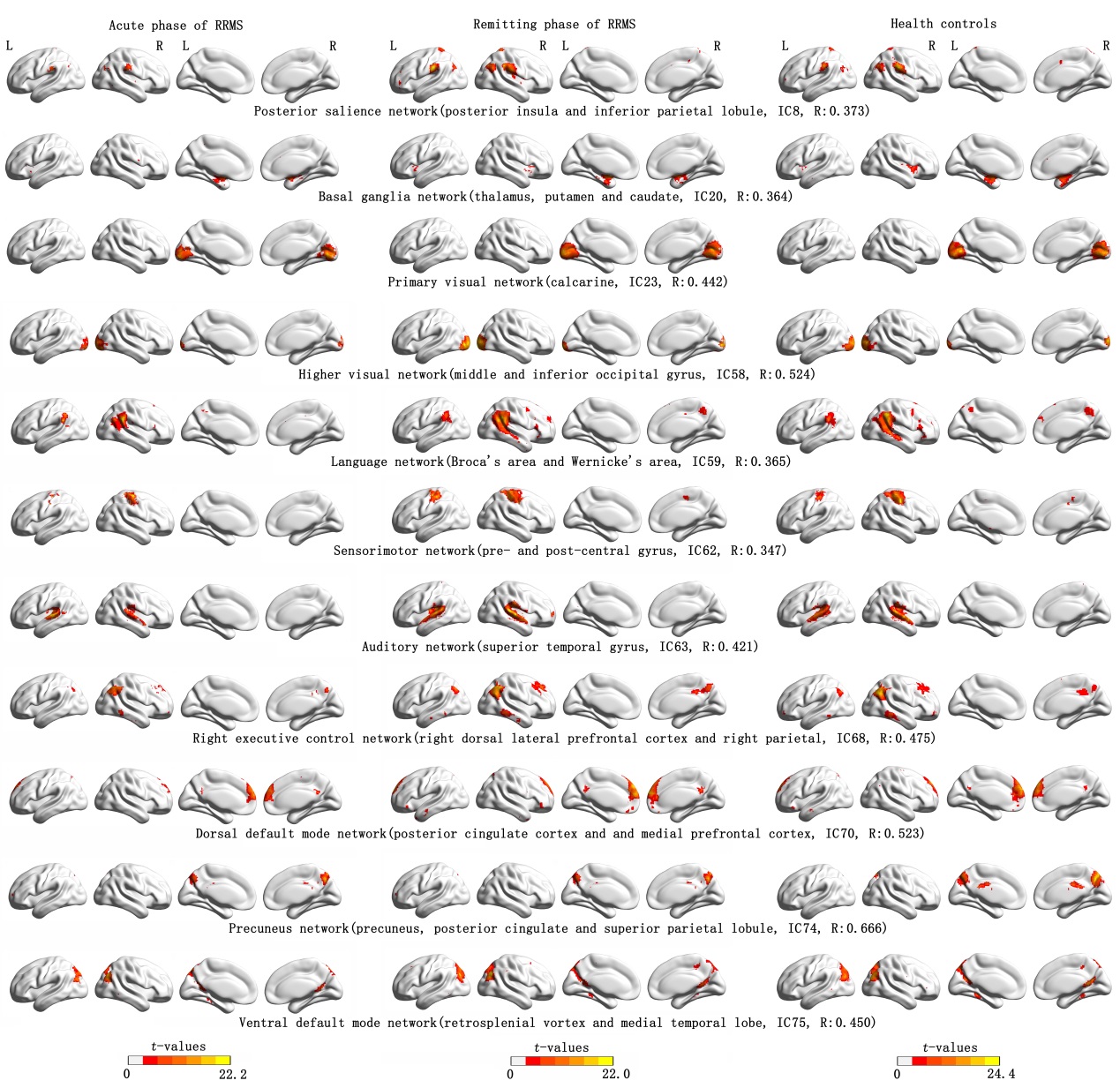


Additional file 1: Fig. S1 Spatial map of the eleven resting state networks from relapsing-remitting multiple sclerosis and health controls. (one-sample *t*-test, P = 0.001, FDR correction).

Note: IC x: independent component x obtained by group independent components analysis. R is the highest correlation coefficient which results from the spatial match of the independent components with the offering templates.


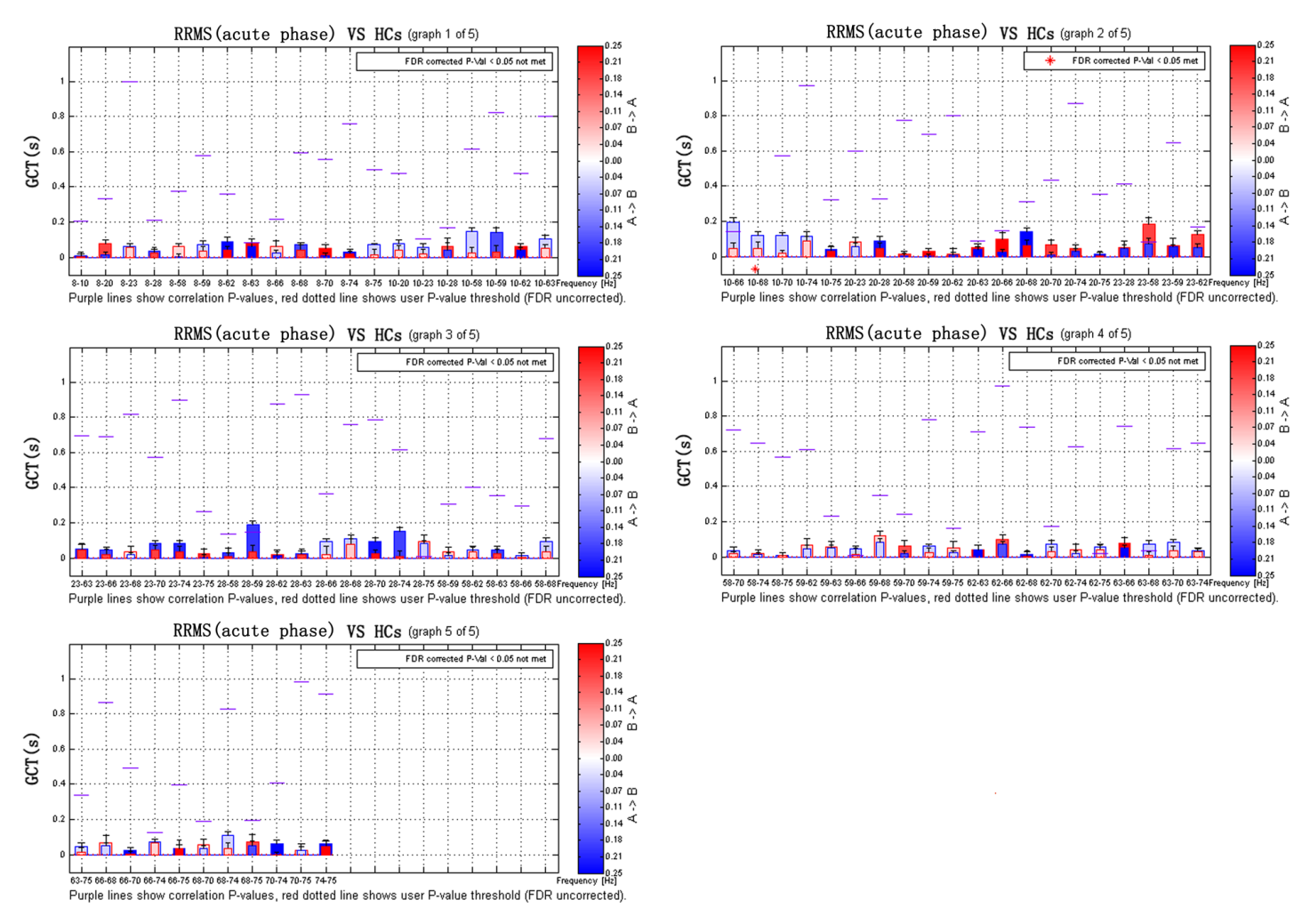


Additional file 1: Fig. S2 Group differences in the GCA between the acute phase RRMS patient and HCs. (P < 0.05, FDR correction).

Note: A> B means component A is larger than component B, eg, in the bar of 10-68, cold-toned (blue) means component 10 is larger than component 68, and warm-toned (red) means component 68 is larger than component 10 in frequency (Hz) of GCT analyses.
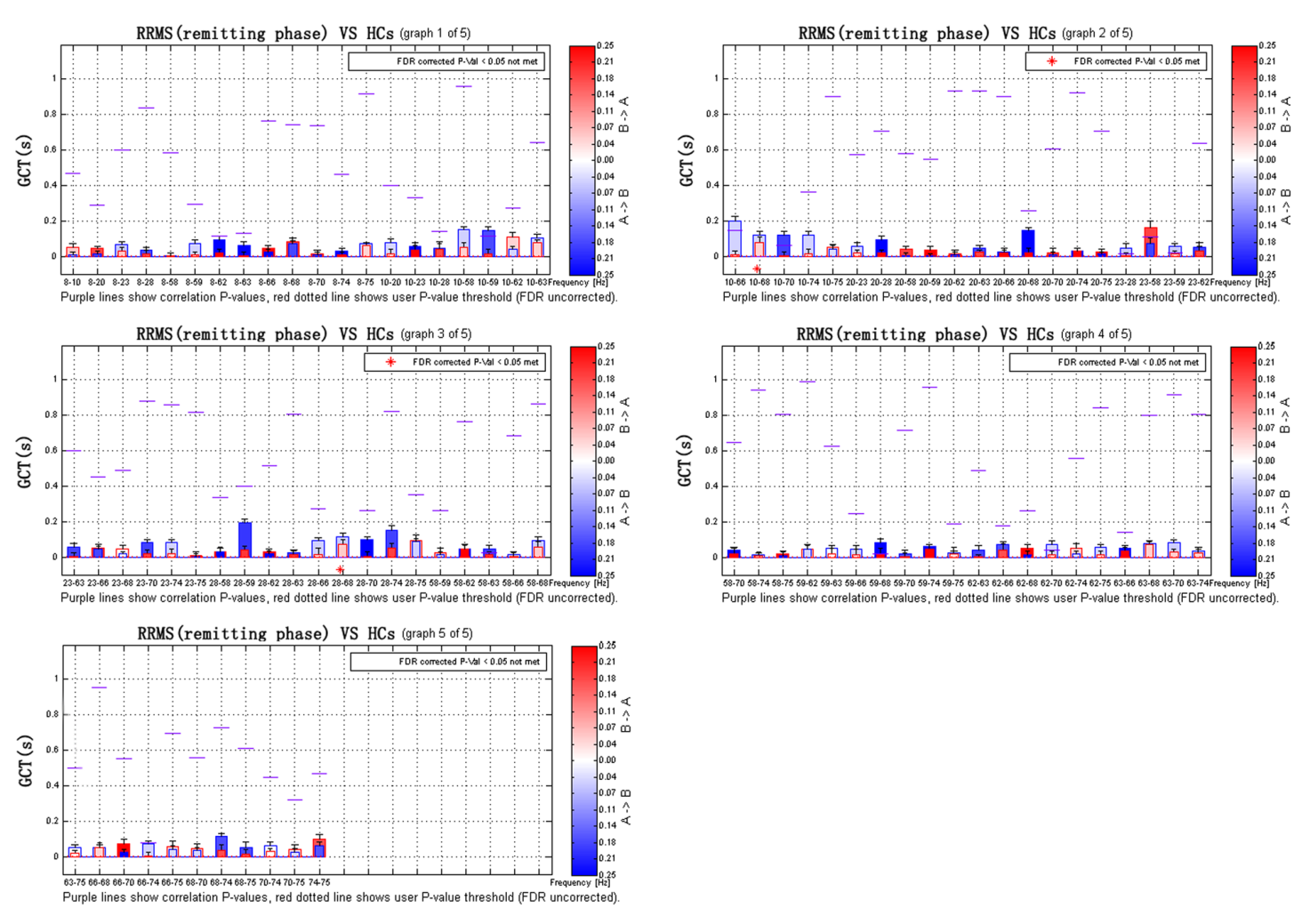


Additional file 1: Fig. S3 Group differences in the GCA between the remitting phase RRMS patient and HCs. (P < 0.05, FDR correction)
